# Between two stools? Pharmacologists nominated for Nobel prizes in “physiology or medicine” and “chemistry” 1901–1950 with a focus on John Jacob Abel (1857–1938)

**DOI:** 10.1007/s00210-020-01993-0

**Published:** 2020-10-15

**Authors:** Michael Pohar, Nils Hansson

**Affiliations:** Faculty of Medicine, Department for the History, Philosophy, and Ethics of Medicine, Heinrich Heine University Dusseldorf, Moorenstr. 5, 40225 Dusseldorf, Germany

**Keywords:** Artificial kidney, Epinephrine, Excellence in pharmacology, John Jacob Abel, Nobel prize, Pharmacology, Posterior pituitary hormones

## Abstract

Since the early stages of its academic professionalization, pharmacology has been an interdisciplinary field strongly influenced by the natural sciences. Using the Nobel Prize as a lens to study the history of pharmacology, this article analyzes nominations of pharmacologists for two Nobel Prize categories, namely “chemistry” and “physiology or medicine” from 1901 to 1950. Who were they? Why were they proposed, and what do the Nobel dossiers say about excellence in pharmacology and research trends? This paper highlights the evaluation of “shortlisted” candidates, i.e., those candidates who were of particular interest for the members of the Nobel Committee in physiology or medicine. We focus on the US scholar John Jacob Abel (1857–1938), repeatedly referred to as the “Founder of American Pharmacology.” Nominated 17 times in both categories, Abel was praised by his nominators for both basic research as well as for his influential positions as editor and his work as chair at Johns Hopkins University. The Abel nominations were evaluated for the Nobel Committee in chemistry by the Swedish professor of chemistry and pharmaceutics Einar Hammarsten (1889–1968), particularly interested in Abel’s work on hormones in the adrenal glands and in the pituitary gland. Eventually, Hammarsten did not view Abel’s work prizeworthy, partly because other scholars had done—according to Hammarsten—more important discoveries in the same fields. In conclusion, analyses of Nobel Prize nominations help us to better understand various meanings of excellence in pharmacology during the twentieth century and beyond.

## Introduction

Since the early stages of its academic professionalization, pharmacology has been an interdisciplinary field strongly influenced by the natural sciences (Phillippu 2018; Starke 1998). While the German pioneers of pharmacology—Rudolf Buchheim (1820–1879), Oswald Schmiedeberg (1838–1921), and Bernhard Naunyn (1839–1925)—had studied medicine, several of their close colleagues had earned a PhD instead of a MD. Using the Nobel Prize as a lens to study the history of pharmacology, the aim of this paper is to take a closer look at pharmacologists nominated for two Nobel Prize categories, namely, for “chemistry” and for “physiology or medicine.” Who were they? Why were they proposed? What was deemed excellence in pharmacology in a Nobel context from 1901 to 1950?

Four scientists have been awarded two Nobel Prizes: Marie Curie (1867–1934), Linus Pauling (1901–1994), John Bardeen (1908–1991), and Frederick Sanger (1908–2013), the latter relevant also for pharmacology since he was awarded his first Nobel Prize in chemistry “for his work on the structure of proteins, especially that of insulin.” This group of double laureates is well known, but not much research has looked into scholars who were nominated for more than one Nobel Prize category (Gross and Hansson 2020). None of the double laureates have so far both “physiology or medicine” and “chemistry” Nobel medals.

Our research group has previously traced Nobel Prize nominees by analyzing nominations and Nobel committee evaluations within the category “physiology or medicine,” e.g., in surgery (Hansson et al. 2019), cardiology (Drobietz et al. 2020), and neurology (Hansson et al. 2020) to investigate the attribution of credit in medicine. In a previous paper, we provided an overview of the “Nobel population of pharmacologists,” i.e., nominees and nominators for the Nobel Prize in physiology or medicine (Pohar 2020). These studies showed certain patterns regarding credit allocation in medicine, e.g., that there were strong social ties between the nominee and the nominator, such as between professor and assistant or faculty colleagues. In addition, we reconstructed a shift of “Nobel” hotspots in pharmacology, places where most nominees and nominators worked, from Central Europe to the USA around 1930. Due to his training in Europe and the USA, pharmacologist John Jacob Abel (1857–1938) contributed to this shift. He was nominated 17 times from 1925 to 1939 for two Nobel Prize categories (“physiology or medicine” and “chemistry”). While much has already been written about Abel, the story of his “Nobel career” has not yet been told. How was he portrayed in nominations and in committee evaluations—and why did he never receive the prize? Furthermore, we intend to show the importance of pharmacology in the context of the Nobel Prize, based on the evaluation of “shortlists,” i.e., those candidates who were of particular interest for the members of the Nobel Committee in physiology or medicine.

Which Nobel fields in science and medicine are trending over time? In a recent paper, Ioannidis et al. (2020) suggested that most Nobel Prizes in physiology or medicine, chemistry, and physics from 1995 to 2017 could be attached to only few scientific domains such as particle physics, cell biology, atomic physics, neuroscience, and molecular chemistry. Reviewed in a longer period of time, we argue that pharmacology, too, has been a major Nobel field since the inception of the Nobel Prize. So far, 13 prizes have strong ties to this discipline in the category physiology or medicine, but these point at only a fraction of the nominated pharmacologists. There are several reasons why promising nominees never received the prize. Science historian, Robert Marc Friedman (2001), analyzed the influence behind the scenes for the Nobel Committee in chemistry. Friedman referred to Svante Arrhenius (1859–1927, Nobel Prize laureate in 1903), who used his influence in the Nobel committee to prevent the awarding of prizes in chemistry to Walther Nernst (1864–1941). Nernst was nominated 58 times in the years 1906–1921. Friedman also showed that one committee member, Ludwig Ramberg (1874–1940), opposed the award of biochemical achievements in the chemistry category.

## Methods

In a previous article, we isolated the group of pharmacologists using the Nobel Prize database with its 5110 nominations in the category physiology or medicine from 1901 to 1953 (Pohar 2020). This article compares this group with nominated pharmacologists for the prize category chemistry from 1901 to 1950, including pharmacologists who were nominated for both categories. We then compared the nominations of the double nominees in order to draw conclusions about trends in pharmacology research and focused on shortlisted nominees to explore what was considered to be excellent research in the context of the Nobel Prize.

The article is based on John Jacob Abel’s Nobel Prize nominations and special investigations, his own publications, and secondary literature. These nominations dossiers were provided through collaboration with Prof. Karl Grandin, Stockholm, Director of the Center for History of Science at the Royal Swedish Academy of Sciences. We also reviewed the Nobel Prize database “nobelprize.org” with the directory of all nominations in chemistry and physiology or medicine.

## Results: “Nobel” networks in pharmacology

Reviewing nomination dossiers of the Nobel committee for chemistry and the Nobel committee for physiology or medicine, we found—next to Abel—several scholars who were nominated for both prize categories, including Nobel laureates such as the previously mentioned Svante Arrhenius, but also Emil Fischer (1852–1919), Eduard Buchner (1860–1917), Paul Ehrlich (1854–1915), Albrecht Kossel (1853–1927), Fritz Pregl (1869–1930), Adolf Windaus (1876–1959), Otto Warburg (1883–1970), Hans Fischer (1881–1945), and candidates who never received the prize such as Fritz Kögl (1897–1959), Rudolf Schoenheimer (1898–1941), Gustav Embden (1874–1933), Choh Hao Li (1913–1987), Jacques Tréfouël (1897–1977), Emil Abderhalden (1877–1950) (Halling et al. 2018), Sachachiro Hata (1873–1938), Sören Sörensen (1868–1939), and Carl Neuberg (1877–1956). They had networks both in chemistry and the life sciences and nominators who emphasized their contributions to both juries to boost their Nobel Prize chances. Several of the proposed pharmacologists during the first half of the twentieth century worked on topics in the gray zone between chemistry and physiology or medicine (Table 1).

**Table 1:** Nominated pharmacologists nominated for the Nobel Prize in chemistry and in physiology or medicine

Name	Number of nominations inchemistry and years	Number of nominationsin physiology or medicine and years	Nobel Prize laureate
John Jacob Abel (1857–1938)	5 (1925–1927)	17 (1925–1939)	
Edward Calvin Kendall (1886–1972)	1 (1949)	27 (1922–1950)	Physiology or medicine, 1950
Jacques Tréfouël (1897–1977)	9 (1940–1951)	3 (1938–1948)	
Sir Hans Adolf Krebs (1900–1981)	7 (1946–1950)	16 (1946–1953)	Physiology or medicine, 1953
Edward Joseph Conway (1894–1968)	2 (1950–1959)	1 (1949)	
Tadeus Reichstein (1897–1996)	15 (1943–1950)	13 (1950–1951)	Physiology or medicine, 1950
Lyman Craig (1906–1974)	28 (1952–1965)	1 (1950)	
Bernard Naftali Halpern (1904–1978)	1 (1966)	2 (1951)	

## John Jacob Abel (1857–1938): A biographical note

**Fig. 1:**
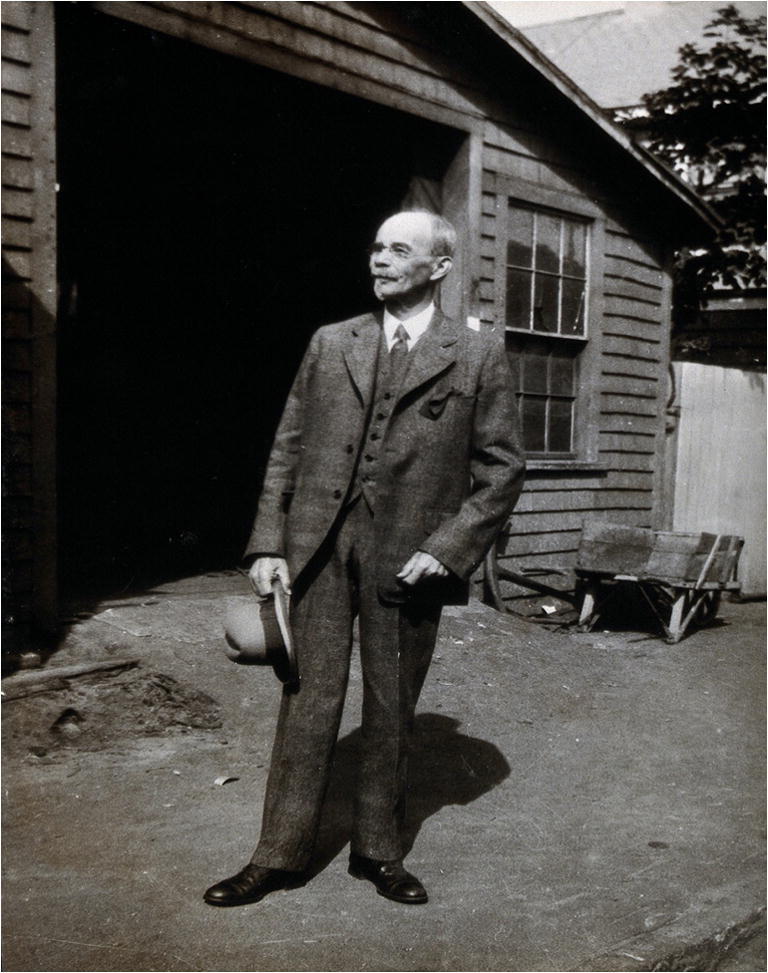
Photograph of John Jacob Abel (Source: Credit: John Jacob Abel. Credit: Wellcome Collection. Attribution 4.0 International (CC BY 4.0))

John Jacob Abel was born into a family of German origin who migrated to the USA in the early 1850s (George and Eknoyan 2012) (Fig. 1). After graduating from the University of Michigan, he briefly worked as a principal of a high school and of the public schools at La Porte, IN. Abel earned a PhD in 1883 (University of Michigan) and then received postdoc training under the physiologist H. Newell Martin (1884–1896) (Marshall 1926) for 1 year in the Biology Department at Johns Hopkins University in Baltimore.

From 1884 to 1891, J. J. Abel traveled to Central Europe to study medicine and chemistry. During these “Wanderjahre,” he had several renowned teachers such as in Leipzig: Carl Ludwig (1816–1895) (physiology), Rudolf Boehm (1844–1926) (pharmacology); in Strassburg: Adolf Kussmaul (1822–1092) (medicine), Bernhard Naunyn (1839–1925) (pathology), and Oswald Schmiedeberg (1838–1921) (pharmacology); in Heidelberg: Vincenz Czerny (1842–1916) (surgery) (Hansson and Tuffs 2016); and in Vienna: Hermann Nothnagel (1841–1095) (medicine) (George 1998). Some of these international contacts, first and foremost Schmiedeberg, had a major impact on his career.

In 1888, Abel was awarded the MD (Dr. med.) by the Kaiser Wilhelm University in Strassburg (now Strasbourg). In January 1891, Abel returned to North America, where he was offered the first full professorship of pharmacology in the USA at the University of Michigan at the recommendation of Oswald Schmiedeberg. In 1893, Abel left Michigan University and was appointed chair in pharmacology at the medical school at Johns Hopkins University, where he worked until his retirement 33 years later in 1932 (until his death in 1938, he continued to serve as director of endocrinological research). It has been put forward that Abel helped many of his students and assistants to important positions in medicine and science, including Reid Hunt (1870–1948) (professor of pharmacology, Harvard Medical School), Carl Voegtlin (1879–1960) (professor of pharmacology Johns Hopkins Medical School), Henry Gray Barbour (1886–1943) (professor of pharmacology, Yale University, Montreal University, University of Louisville), and Eli Kennerly Marshall (1889–1966) (professor of pharmacology, Washington University in St. Louis and Johns Hopkins University). Parascandola (1992) also emphasized that Abel shaped the discipline through his students at other US universities such as PA and Columbia. Therefore, it is not surprising that Abel has repeatedly been described as the “Founder of American Pharmacology” (George 1998), corresponding to the oeuvre of Oswald Schmiedeberg as “Father of modern Pharmacology” (Van Ree and Breimer 2008, but also in a Nobel nomination for Schmiedeberg by Max Cloëtta (1886–1940) as early as in 1910). Abel’s achievements include, for instance, the foundation of the Journal of Experimental Medicine in 1896, the Journal of Biological Chemistry in 1905, and the Journal of Pharmacology and Experimental Therapeutics in 1906. The American Society of Biological Chemists (since 1987 American Society for Biochemistry and Molecular Biology) (ASBMB) in 1909 and the American Society of Pharmacology and Experimental Therapeutics (ASPET) in 1908 were also created under his direction. In 1906, Abel became the first vice president of ASBMB (Kregse 2008), and president of ASBMB in 1908, as well as the first president of ASPET from 1909 to 1912. Next to these gate-keeping positions, Abel had several scientific interests. He is remembered for having contributed to the isolation of epinephrine (Abel 1901; Abel 1903; Abel and Taveau 1904) and insulin (Abel et al. 1925; Abel 1926; Abel et al. 1927; Parascandola 1992) and for work research on posterior pituitary hormones (Abel 1919; Abel and Rouiller 1922; Abel et al. 1923; Abel 1924; Abel 1930). These interests were reflected in the nomination letters for Abel (Table 2).

**Table 2:** Nominations for John Jacob Abel for the Nobel Prize in chemistry and physiology or medicine

Year	Name nominator	Reason for nomination
Nobel Prize nominations for JJ Abel in chemistry		
1925	Charles Walcott	Demonstration of active principle of adrenal gland. Work on pituitary gland. Work on chemotherapy.
1926	Charles Walcott	Demonstration of active principles of adrenal gland, preparation and isolation of active principle. Work on pituitary. Work on chemotherapy.
1927	George Hale	Isolation of insulin; chemical nature of insulin and separation in crystalline from.
1927	Henry Fairfield Osborn	No reason expressed.
1927	James Norris	Study of ductless glands—basic nature of adrenal gland, preparation of the active principle. Work on insulin and crystalline preparation.
1927	Charles Walcott	Demonstration of active principles of adrenal gland, preparation and isolation of active principle. Work on pituitary. Work on chemotherapy.
Nobel Prize nominations for JJ Abel in physiology or medicine		
1925	Hugh McGuigan	Studies of the endocrine glands, and isolation of epinephrine.
1925	Emanuel Libman	Studies of the endocrine glands, and of elective excretion of dyes.
1927	Robert Lowie	Preparation of crystalline insulin.
1928	Charles Russell Bardeen	Notable contributions in the field of hormones.
1930	Charles Mayo	Work on the introduction of epinephrine and the crystallization of insulin.
1930	Emanuel Libman	Work on epinephrine, elective excretion of dye-stuffs, and the nature and identification of the active principle of the pituitary gland.
1931	Ross Gortner	Work on epinephrine, vividiffusion and insulin.
1931	Arthur Douglass Hirschfelder	Work on epinephrine and insulin.
1932	William Ford	Work on the crystallization of insulin.
1932	Eli Kennerly Marshall	Work on the crystallization of insulin.
1934	Benjamin Brecknell Turner	Work on the isolation of hormones (insulin, epinephrin), and the active unitary principle from the posterior lobe of the pituitary body.
1939	Eben James Carey	Work on the isolation, purification and crystallization of the hormones from the glands of internal secretion.

### Media attention and criticism

The work on vividiffusion (dialysis) apparatus and plasmapheresis, a dialysis precursor have been attributed to him, and Abel has been described as the first person who had the idea of passing blood of a living animal through a dialysis membrane to wash out certain substances (George and Eknoyan 2012). This discovery attracted media attention, for example in the London Times (August 11, 1913) and the New York Times (January 18, 1914). Media celebrated Abel’s work as groundbreaking and referred to it as the “Artificial Kidney,” which created headlines like “New Poison Test” (Times, Watertown, NJ), “Artificial Kidney Poison Detective: Professor of Johns Hopkins University invents device to check for suicide” (New York Herald), “Reveals Poisons in Blood” (New York Evening Post), “The Artificial Kidney” (New York Times), and “La Purification Du Sang” (Le Petit Niçois, Paris) (George 1998). Abel himself objected the term “artificial kidney” but welcomed the description by his German colleague George Haas (1886–1971), who spoke about “dialyzing patients” (George and Eknoyan 2012). Although Abel did not make a breakthrough with the idea and the first clinically successful hemodialysis in a human was only successfully established by Willem Kolff (1911–2009) 30 years later (Gottschalk and Fellner 1997), Abel is considered to have been an indirect influence on Kolff (George and Eknoyan 2012). However, critics accused Abel and his colleagues of non-transparent behavior regarding the publication of precise data. They questioned if the investigated substances were toxic at all and meant that the safety of the dialysis apparatus to use for patients was inadequate. Later, Abel was inspired by the topic of plasmapheresis and started researching it by performing experiments with dogs (George 1998). He concluded that the method clearly improved the conditions of the dogs. He then applied the technique on a female patient, who suffered from complications after the treatment. Observers of the procedure accused Abel later to not maintain adequate hygiene standards and the performed plasmapheresis was described as harmful. After this incident, Eli K. Marshall (1889–1966) from Abel’s laboratory examined the study results and concluded that the safety of this method remained unclear. However, Abel still continued to praise plasmapheresis and dialysis in his lectures, for instance in his Mellon Lecture in February 1915 (George 1998).

### Publications and citations

Abel published more than 50 scientific articles in different journals, predominantly in the Journal of Pharmacology and Experimental Therapeutics (20), where he served as editor for 23 years, and the Bulletin of the Johns Hopkins University (12), with a first authorship in 49 and last authorship in 3 articles. According to Web of Science, Abel’s article citations reached a peak in 1945 with 45 citations that year, but his work is still mentioned on a regular basis: Altogether, his articles were cited more than 250 times between 2010 and 2020 (Web of Science October 1, 2020). Would it have been greater if he had received the Nobel Prize? It still is an open research question whether the canonization as Nobel laureate has a major influence on the number of citations. A previous study on the Nobel laureate John C. Eccles (1903–1997) did not find a clear-cut Nobel effect in the citation pattern (De Sio et al. 2019).

Table 3 lists Abel’s ten most often cited articles. They mirror major topics for which he was proposed for the Nobel Prize (crystalline insulin and research on the pituitary gland), but also work that was not brought up by nominators, such as laxatives, (phthaleins and their behavior as purgatives, 1909) and tetanus. The two to date most cited publications deal with dialysis, both published in 1914. Dialysis (“vividiffusion”) was only once explicitly mentioned in a proposal by Ross Gortner (1885–1942) in 1931 as one of the several motives of nomination. One reason for the relatively high number of citations might have been the media interest at the time. The “top ten” publications include both early and late-career work. One of these articles was published in the year of his death (1938).

**Table 3:** Ten of Abel’s most cited key papers*

	Year	Number of citations	Author	Namepublication	Name journal	Position in journal
1	1914	257	Abel, JJ; Rowntree, LG; Turner, BB	On the removal of diffusiblesubstances from the circulatingblood of living animals by dialysis	Journal of Pharmacology and Experimental Therapeutics	Volume: 5, Issue: 3, Pages: 275–316
2	1914	166	Abel, JJ.; Rowntree, LG; Turner, BB	Plasma removal with return of corpuscles	Journal of Pharmacology and Experimental Therapeutics	Volume: 5, Issue: 6, Pages: 625–641
3	1926	138	Abel, JJ	Crystalline insulin	Proceedings of the National Academy of Sciences of the United States of America	Volume: 12, Pages: 132–136
4	1909	107	Abel, JJ; Rowntree, LG	On the pharmacological action of somephthaleins and their derivatives, withespecial reference to their behavioras purgatives I	Journal of Pharmacology and Experimental Therapeutics	Volume: 1, Issue: 2, Pages: 231–264
5	1919	98	Abel, JJ; Kubota, S	On the presence of histamine(beta-iminazolylethylamine) in thehypophysis cerebri and other tissuesof the body and its occurrence amongthe hydrolytic decomposition productsof proteins	Journal of Pharmacology and Experimental Therapeutics	Volume: 13, Issue: 3, Pages: 243–300
6	1927	76	Abel, JJ; Geiling, EMK; Rouiller, CA; et al.	Crystalline insulin	Journal of Pharmacology and Experimental Therapeutics	Volume: 31, Issue: 1, Pages: 65–85
7	1935	59	Abel, JJ; Evans, EA; Hampil, B; et al.	Researches on tetanus II. The toxin of thebacillus tetani is not transported to thecentral nervous system by any component of the peripheral nerve trunks	Bulletin of the Johns Hopkins Hospital	Volume: 56, Pages: 84–114
8	1938	52	Abel, JJ; Firor, WM; Chalian, W	Researches on tetanus IX. Further evidenceto show that tetanus toxin is not carried to central neurons by way of the axis cylinders of motor nerves	Bulletin of the Johns Hopkins Hospital	Volume: 63, Issue: 6, Pages: 373–403
9	1935	50	Abel, JJ; Hampil, B; Jonas, AF	Researches on tetanus III. Further experimentsto prove that tetanus toxin is not carried inperipheral nerves to the central nervous system	Bulletin of the Johns Hopkins Hospital	Volume: 56, Pages: 317–33
10	1935	48	Abel, JJ; Hampil, B	Researches on tetanus IV. Some historicalnotes on tetanus and commentaries thereon	Bulletin of the Johns Hopkins Hospital	Volume: 57, Issue: 1, Pages: 343–372

*The list is obtained by the access to Web of Science on September 29, 2020

### Nobel prize nominations

Abel’s hormonal research was a key argument in the letter of nomination for the Nobel Prize in chemistry by Charles D. Walcott on November 19, 1925. Walcott added a statement by Dr. Reid Hunt, Department of Pharmacology, Harvard Medical School, to strengthen his line of reasoning:

“... he first demonstrated the basic nature of the active principle of the adrenal gland and prepared a benzoyl derivative of the active principle; this constituted the first isolation of the active principle in pure form. As regards his recent work on the pituitary, he has shown that the various physiological actions are due to a single substance and not to a number as other workers had believed.” (Nobel Committee for Chemistry 1926)

Another milestone in Abel’s research was pointed out in the same proposal:

“... His work with Rowntree on the Chemotherapy of Organic Antimony compounds is the most important which has been done since the original work with tartar emetic*.” (Nobel Committee for Chemistry 1926)

*Tartar emetic: Antimony potassium tartrate; used in schistosomiasis and leishmaniasis because of its emetic action.

Even if the “artificial kidney” caused a lot of media attention, Abel was nominated exclusively for his other achievements for the Nobel Prize in chemistry, as it can also be seen from Georg Hale’s nomination on November 24, 1926:

“... In my judgment, his discovery of the chemical nature of insulin and its separation in the crystalline form would amply justify the award to him ... When his previous work is also taken into account, his position as a leading professor in physiological chemistry becomes apparent...” (Nobel Committee for Chemistry 1927)

In another nomination by Charles D. Walcott on December 17, 1926, Walcott listed the arguments in his previous nominations again. He focused on research on the adrenal gland, pituitary and work on chemotherapy, expanding his arguments with a new argument:

“..... He also discovered methods by which more selective preparations can be secured than had been obtained previously….” (Nobel Committee for Chemistry 1927)

This time, Walcott attached a list of “the titles of Doctor Abel’s papers.”

**Fig. 2:**
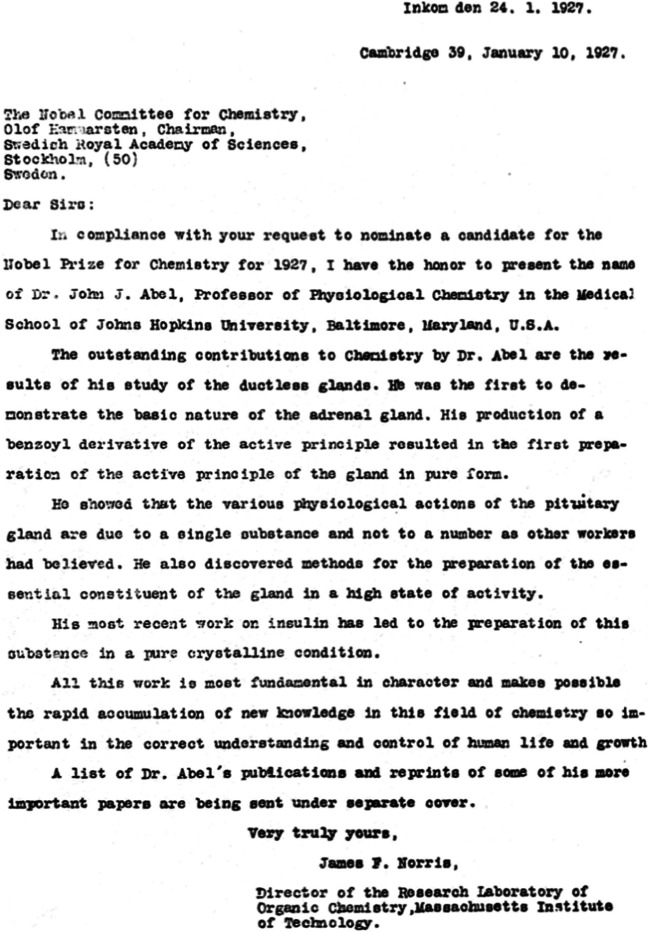
Nobel Prize nomination for JJ Abel by James F Norris in 1927. (Credit: Royal Academy of Sciences, Stockholm)

Another nomination by James F. Norris, the former President of the American Chemical Society (1925–1926) and then acting Vice President of the International Union of Pure and Applied Chemistry (1925–1928) can also be found in the Nobel Prize Archives (Fig. 2). Norris took up the same arguments from Walcott’s nomination from the 1927 yearbook. He ended with the summarizing statement:

*“... All this work is most fundamental in character and makes possible the rapid accumulation of new knowledge in the field of chemistry so important in the correct understanding and control of human life and growth…”* (Nobel Committee for Chemistry 1927)

As stated in Alfred Nobel’s last will, the Nobel Prizes shall be awarded for work “of the greatest benefit to mankind.” The Nobel committee has clarified that a candidate—at least in physiology or medicine—is not supposed to be awarded for a lifetime achievement, but for a single important discovery: “prizes are thus given for specific scientific achievements rather than for general merit in medical research” (Liljestrand 1962). Thus, nominators that proposed Abel for activities, as ‘science manager’ and underlined that he had established scientific societies and journals were counterproductive.

### Verdict by the Nobel committee

Abel’s nomination in 1926 gave rise to an in-depth analysis by Nobel Prize committee member, the Swedish professor of chemistry and pharmaceutics, Einar Hammarsten (1889–1968), who worked at the Karolinska Institute from 1928 to 1957. This kind of special investigation was made for potentially prizeworthy achievements. Of particular interest to Hammarsten was Abel’s work on hormones in the adrenal glands and in the pituitary gland. Regarding adrenalin, or epinephrine as Abel called it, the first hormone that was produced in pure form, Hammarsten was not enthusiastic. He wrote that Abel’s research was quite old (“conducted more than twenty years ago”), and that Abel was not the first to isolate adrenalin, but the Japanese chemist Jokichi Takamine (1854–1922) and Thomas Aldrich (1861–1938) in 1901. Furthermore, Hammarsten continued, Friedrich Stolz (1860–1936) and Ernst Joseph Friedmann (1877–1957) had from 1904 to 1906 also made significant discoveries with regard to the constitution and synthesis of adrenalin. Therefore, Hammarsten did not view Abel’s work as truly pioneering, and he reached the same conclusion regarding his research on hormones in the pituitary gland. Hammarsten’s verdict: “The result of my review of Abel’s research on hormones is that it is not of the greatest importance and it has not been carried out on an outstanding scientific level, so that I cannot recommend Abel for a Nobel Prize in chemistry.” Instead, the Nobel committee agreed on the Austrian scholar Richard Adolf Zsigmondy (1865–1929) “for his demonstration of the heterogeneous nature of colloid solutions and for the methods he used, which have since become fundamental in modern colloid chemistry.”

Although he never received the Nobel Prize, he got several other medals and awards, such as the Gold Medal (Society of Apothecaries) London in 1928, the Conné Medal, New York Chemists’ Club in 1932, and the Kober Medal in 1934 (MacNider 1946).

As mentioned above, Abel was far from the only pharmacologist who was evaluated by the Nobel committee. Table 4 shows the pharmacologists that were shortlisted in 1901–1949. The relatively high number of pharmacologists listed shows the importance pharmacology had in the context of the Nobel Prize.

**Table 4:** Evaluated pharmacologists by the Nobel committee for physiology or medicine between 1901 and 1949 and reasons for nomination of candidates on shortlist

Year	Pharmacologist	Name of nominator	Reason for nomination	Nobel Prize laureate (links to pharmacology)
1902	Ernest Overton (1865–1933)	Justus Gaule (1849–1939)	Work on osmosis in plantand animal cells, and on anesthesia.	
1906	Ernest Overton (1865–1933)	Justus Gaule (1849–1939)	Work on the osmosis inmuscles and nerves.	
1922	Edward Calvin Kendall (1886–1972) Rudolf Magnus (1873–1927)	Kendall: Richard Zeynek (1869–1845) Magnus: Ernst Laqueur (1880–1947)	Kendall: The purification ofthyroxine and description of its structure. Magnus: Work on the nervous system and in thearea of the mechanics of the intestines.	
1927	Rudolf Magnus (1873–1927) Otto Loewi (1873–1961)	Magnus: Sir Archibald V Hill (1886–1977) Alfred Denker (1863–1941) John James Macleod (1876–1935) Martin Kochmann (1878–1936) Phillipp Stöhr (1891–1979) Adolf Jarisch (1891–1965) Hugo Winternitz (1868–1934) Loewi: Ernst Willhelm von Brücke (1880–1941) Adolf Jarisch (1891–1965)	Magnus: Studies of posture and its dependence on the labyrinths and proprioceptive pathways from muscles and joints. Work on posture, especially as described in his work “Die Körperstellung” (J. Springer, Berlin, 1924). Work on posture, muscle tonus and tonic reflexes. Work on posture, muscle tonus and tonic reflexes. Work on posture, muscle tonus and tonic reflexes. Studies of posture and its dependence on the labyrinths and proprioceptive pathways from muscles and joints. Work on posture, muscle tonus and tonic reflexes. Loewi: Discovery of an hormonal system controlling action of the heart. Chemical transmission of nerve impulses in the heart.	
1934	Corneille Jean François Heymans (1892–1968)	Arnold During (1872–1961) Paul John Hanzlik (1885–1951)	Work on the regulation of respiration and blood circulation. Demonstration that the reflexes from the trunk and head are physiologically important in the control of respiration and circulation; and the importance of these reflexes in pharmacological reactions.	
1936	Otto Loewi (1873–1961) Sir Henry Hallett Dale (1875–1968)*, Corneille Jean François Heymans (1892–1968)	Loewi: Robert John Steward McDowall (1892–1990) Ernst Willhelm von Brücke (1880–1941) Emil Starkenstein (1884–1942) Hans Horst Meyer (1853–1939) Sir Henry Hallett Dale (1875–1968) George Barger (1878–1939) Adolf Jarisch (1891–1965) Gunnar Ahlgren (1898–1962) Sven Ingvar (1889–1947) Heymans: B T Krishnan	Loewi: Work on humoral transmission of nervous impulses to tissues. Work on the humoral transmission of nervous impulses. Work on humoral transmission of nervous impulses to tissues. Work on the humoral transmission of nervous impulses. Work on the humoral transmission of nervous impulses. The discovery of the humoral transmission to the heart from the vagus nerve. Work on humoral transmission of nervous impulses to tissues. Work on the humoral transmission of nervous impulses, Work on the humoral transmission of nervous impulses. Heymans: Work on the influence of sinus caroticus on the rate of the heartbeat, and on blood pressure and respiration.	Loewi, Dale: For their discoveries relating to chemical transmission of nerve impulses.
1939	Gerhard Domagk (1895–1964) Sir Edward Mellanby (1884–1955) Alfred Newton Richards (1876–1966)	Domagk: Arthur Duncan Gardner (1884–1977) Mellanby: Stevenson Lyle Cummins (1873–1949) Sir Frederick G Hopkins (1861–1947) Sir Charles S Sherrington (1857–1952) Joshua Harold Burn (1892–1981) Richards: Donald D van Slyke (1883–1971) Sir Archibald V Hill (1886–1977)	Domagk: Discovery of the antibacterial effects of Prontosil. Mellanby: Work on dietary deficiencies, rickets and the nervous conditions produced by lack of vitamin A. Rickets and the nervous conditions produced by lack of vitamin A. Rickets and the nervous conditions produced by lack of vitamin A. Rickets and the nervous conditions produced by lack of vitamin A. Richards: Work on the physiology of the kidneys (mechanism of renal secretion). Work on the physiology of the kidneys.	Domagk: For the discovery of the antibacterial effects of prontosil.
1940	Alfred Newton Richards (1876–1966)	Paul Govaerts (1889–1960)	Physiology of the kidney.	
1941	Edward Calvin Kendall (1886–1972)	Norman M Keith (1885–1976) Willis S. Lemon (1878–1954) Frank C Mann (1887–1962) George B Eusterman (1882–1966)	Work on the chemical composition and physiologic action of the hormones of the adrenal cortex Work on the chemical composition and physiologic action of the hormones of the adrenal cortex. Work on the chemical composition and physiologic action of the hormones of the adrenal cortex. Work on the chemical composition and physiologic action of the hormones of the adrenal cortex.	
1945	Alfred Newton Richards (1876–1966)	W N Bradley Alexander Randall (1883–1951) Carl F Schmidt (1893–1988) Edward B. Kumbhaar (1882–1966) Walter G Elmer (1872–1960) Henry C Bazett (1885–1950) Isaac Starr (1895–1989)	Assisting materially in the development of the production of penicillin. Work on the physiology of the kidneys. Assisting materially in the development of the production of penicillin. Richards was also nominated (in the same letter) for his work in spreading the use of pennicillin. Work on the physiology of the kidneys. Work on the physiology of the kidneys. Work on the physiology of the kidneys. Work on the physiology of the kidneys. Richards was also nominated (in the same letter) for his work in spreading the use of penicillin.	
1949	Edward Joseph Conway (1894–1968) Walter Kikuth (1896–1968) Hans Mauß (1901–1953) Fritz Mietzsch (1896–1958)	Joseph F Donagan (1893–1972) Kikuth: Otto Krayer (1899–1982) Mauß: Otto Krayer (1899–1982) Mietzsch: Otto Krayer (1899–1982)	Work on the permeability of the cell wall for ions, and ionic equilibrium. Kikuth: Discovery of Atabrin (quinocrin hydrochloride), an antimalarial agent. Mauß: Discovery of Atabrin (quinocrin hydrochloride), an antimalarial agent. Mietzsch: Discovery of Atabrin (quinocrin hydrochloride), an antimalarial agent.	

*Laureate who worked on the same topic as the pharmacologists but were not pharmacologists by profession

In order to be able to deduce which research topics that were of particular importance, we listed the reasons for nomination in the year of the shortlist (Table 4). The double nominees were proposed between 1925 and 1939.

## Discussion: Culture of remembrance

Abel is remembered for acting as a hub in the international scientific community in the field of pharmacology even after his retirement: On the day of his death, he was elected member of the Royal Society (George 1998). Several scholars payed tribute to Abel and his works, for instance with the presentation of a biographical memoir at the annual meeting of the National Academy of Sciences of the USA in 1946 (MacNider 1946) or in a celebration in 1957 due to the centennial of the Society of Pharmacology and Experimental Therapeutics. This took place in the form of an exhibition containing Abel letters, notebooks, and various articles. At this exhibition laboratory notes about the first isolation of epinephrine from its benzoyl derivate, the chemical isolation of crystalline insulin and recordings of his “artificial kidney” were made available to the public, and pictures of his laboratory colleagues were shown (Marshall 1958). In the same year, an article was published in JAMA summarizing Abel’s life and oeuvre, ending with “Dr. Abel’s contributions to the development of basic medical science in America were truly outstanding, and his influence on contemporary workers was lasting and profound. Practicing physicians may well join with their academic colleagues in paying homage to this great scientist and teacher.” (NN 1957).

In 2008, a game called “What is your Abel number” was played at the celebration of ASPET’s centennial (Parascandola 2007). Here, ASPET members tried to be as closely related to John Jacob Abel as possible in a ranking of numbers from 1 to 6. Furthermore, the John Jacob Abel Award (first awarded in 1947) is annually given to scientists who have been recognized for excellence in pharmacology.

This paper shows that Abel was the first scholar nominated for the two Nobel Prize categories chemistry and physiology or medicine. He is an ideal example to illustrate the close ties of pharmacology between two categories in a Nobel Prize setting. In the end, he was not deemed prize-worthy in either category. He embodied another kind of scientific excellence, attributed to him in different contexts, ranging from eponymous discoveries like the description of the principle of the adrenal gland, his work on pituitary hormones, and on insulin, his more than 30-year career as full professor at prestigious universities, and founder of journals like the Journal of Experimental Medicine, the Journal of Biological Chemistry, and the Journal of Pharmacology and Experimental Therapeutics.
